# The causal relationship between inflammatory bowel diseases and erythema nodosum: a bidirectional two-sample mendelian randomization study

**DOI:** 10.1186/s12876-024-03330-8

**Published:** 2024-07-23

**Authors:** Min Zhong, Hongjin An, Huatian Gan

**Affiliations:** 1 Department of Gastroenterology and the Center of Inflammatory Bowel Disease, West China Hospital, Sichuan University, 37 Guoxue Road, Wuhou District, Chengdu, 332001 Sichuan China; 2 Department of Geriatrics and National Clinical Research Center for Geriatrics, West China Hospital, Sichuan University, Chengdu, China; 3 Department of Gastroenterology and Laboratory of Inflammatory Bowel Disease, the Center for Inflammatory Bowel Disease, Clinical Institute of Inflammation and Immunology, Frontiers Science Center for Disease-related Molecular Network, West China Hospital , Sichuan University, Chengdu, China

**Keywords:** Inflammatory bowel diseases, Crohn’s disease, Ulcerative colitis, Erythema nodosum, Mendelian randomization

## Abstract

**Background:**

Individuals with inflammatory bowel disease (IBD) exhibit a heightened likelihood of developing erythema nodosum (EN), but the presence of causal link is unknown. The purpose of the present research was to investigate this connection using a bidirectional two-sample Mendelian randomization (MR) analysis.

**Methods:**

Summarized statistics for EN were sourced from the FinnGen consortium of European ancestry. The International Inflammatory Bowel Disease Genetic Consortium (IBDGC) was used to extract summary data for IBD. The inverse variance weighted (IVW) technique was the major method used to determine the causative link between them.

**Results:**

The study evaluated the reciprocal causal link between IBD and EN. The IVW technique confirmed a positive causal link between IBD and EN (OR = 1.237, 95% CI: 1.109–1.37, *p* = 1.43 × 10^− 8^), as well as a strong causality connection between Crohn’s disease (CD) and EN (OR = 1.248, 95% CI: 1.156–1.348, *p* = 1.00 × 10^− 4^). Nevertheless, a causal connection between ulcerative colitis (UC) and EN could not be established by the data. The reverse MR research findings indicated that analysis indicated that an increase in EN risks decreased the likelihood of UC (OR = 0.927, 95% CI: 0.861–0.997, *p* = 0.041), but the causal association of EN to IBD and CD could not be established.

**Conclusion:**

This investigation confirmed that IBD and CD had a causal connection with EN, whereas UC did not. In addition, EN may decrease the likelihood of UC. Further study must be performed to uncover the underlying pathophysiological mechanisms producing that connection.

**Supplementary Information:**

The online version contains supplementary material available at 10.1186/s12876-024-03330-8.

## Introduction

The term “inflammatory bowel disease” (IBD) refers to a group of chronic immune-mediated intestinal illnesses that include Crohn’s disease (CD) and ulcerative colitis (UC) [1]. Typical clinical symptoms of IBD include abdominal pain, diarrhea, chronic stomach pain, fatigue, and weight loss [2–4]. In addition, IBD may present with several kinds of extraintestinal manifestations (EIMs) that impact multiple systems, primarily the musculoskeletal, skin, and eyes [5]. According to reports, the frequency of EIMs in IBD patients varies between 6–47% [6–11], with approximately a quarter of patients experiencing EIMs prior to being diagnosed with IBD [12]. EIMs dramatically raise the disease burden and negatively impact the quality of life (QoL) of IBD patients, often even more so than the intestinal illness itself.

A clinically prevalent recurring panniculitis, erythema nodosum (EN) is typified with painful subcutaneous nodules and non-ulcerative erythema around the front part of both shins [13]. Systemic symptoms like fever, headaches, and gastrointestinal issues are often present in conjunction with the skin lesions [14]. Although the precise etiology of EN is yet unknown, the most frequent causes of EN are infections caused by streptococci, primary tuberculosis, sarcoidosis, Behcet’s disease, medicines, pregnancy, and IBD [13, 15, 16]. About 4–15% of people with CD and 3–10% of those with UC experience EN, the most prevalent cutaneous symptom of IBD [17]. In most of the research, EN develops following an IBD diagnosis. However, a sizable cohort study found that 14.3% of individuals had an EN diagnosis prior to an IBD diagnosis [12]. IBD and EN have certain genetic, environmental, and pathophysiology variables in common [18]. However, there isn’t any proof linking this correlation to causality. In terms of therapy and medicine, causation is more valuable clinically than association. there is no evidence to attribute this association to causation.

Mendelian randomization (MR), a genetic epidemiology technique, has gained popularity as a helpful instrument for assessing the causality between exposures and outcomes [19]. This strategy avoids confounding variables and reverse causation by taking advantage of the random allocation of genetic variation, much like a randomized controlled trial (RCT) [20]. Using statistics from the extensive genome-wide association study (GWAS), our research used a two-sample MR analysis to ascertain the causal link between IBD, its subtypes (CD and UC) and EN.

## Materials and methods

### Study design

**Fig. 1:**
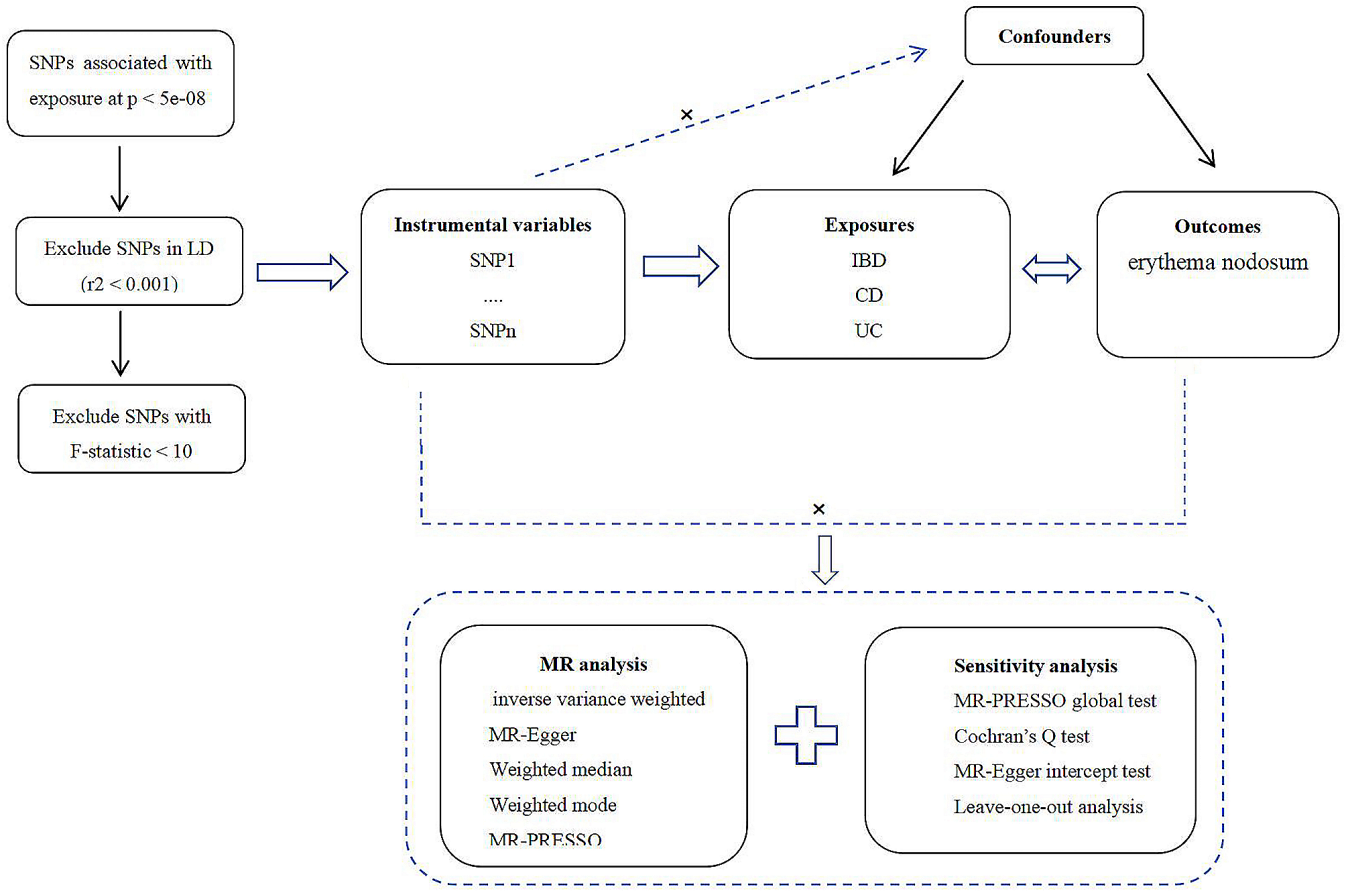
Schematic overview of the present study. SNPs, single-nucleotide polymorphisms; LD: linkage disequilibrium; IBD, inflammatory bowel disease; EN, erythema nodosum; CD, Crohn’s disease; UC, ulcerative colitis; IVW: Inverse variance weighted; MR-PRESSO, MR Pleiotropy residual sum and outlier

For the MR investigation, several single nucleotide polymorphisms (SNPs) were chosen as instrumental variables (IVs). Three key presumptions form the foundation of the MR method (Fig. 1). First, IVs should be strongly connected to the exposures. Second, confounders should not be associated with IVs. Third, rather than via other routes, IVs should impact outcome risk by exposures. The present MR analysis was built on summary data from ethically approved published studies, no additional approval is needed.

### Data sources

The FinnGen Consortium provided summary statistics of EN, comprising 904 cases and 398,204 controls. EN was diagnosed using the ICD-10 (International Classification of diseases) criteria. The summary data of IBD were from the International Inflammatory Bowel Disease Genetics Consortium (IIBDGC) and contained 12,882 IBD cases and 21,770 controls, 6,968 UC cases and 20,464 controls, and 5,956 CD cases and 14,927 controls. IBD and its subgroups were diagnosed using established endoscopic, histological, and radiological criteria. There were no overlapped populations between the exposures and the results.

### Selection of instrumental variables

A variety of inspection methods were applied for the screening of qualified IVs. IVs for IBD (CD or UC) were screened at a genome-wide significance level (*p* < 5 × 10^− 8^). Furthermore, we established *p* < 5 × 10^− 6^ as the screening requirement for IVs in EN. To guarantee the independence of SNPs for IBD, the SNPs with linkage disequilibrium (LD) value (r2 > 0.001) were removed. SNPs having a F value (F = β^2^/SE) greater than 10 were selected to reduce instrument bias. Then, selected SNPs linked to confounding factors were deleted by scanning PhenoScannerV2 for potential associations, such as smoking, celiac disease, and systemic lupus erythematosus [21, 22]. Both exposure and result data were harmonized to make sure that each IV had been matched to the same effect alleles. Moreover, outlier SNPs were removed based on MR pleiotropy residual sum and outlier (MR-PRESSO) analysis to achieve a consistent outcome.

### Immunosuppressants analysis

To investigate the effect of immunosuppressants use on the risk of EN in patients with IBD, GWAS statistics on immunosuppressants use were collected from the UK Biobank, comprising 3,954 cases and 268,648 controls [23]. Instrumental variables for immunosuppressants were selected based on specific parameters, such as *P* < 5 × 10 − 8, r2 < 0.001 and distance = 10,000 kb. MR analysis was then employed to investigate the potential causal relationship between immunosuppressants use and EN. In the event of detecting a significant causal relationship, SNPs closely linked to immunosuppressants would be selectively excluded. Similarly, an MR study employed this approach to investigate the impact of immunosuppressants on the risk of herpes virus infection in patients with IBD [24].

### Statistical analyses

MR analysis was performed in two directions. First, we examined the causal relationship of IBD to EN, and then we investigated the causal outcome of EN to IBD. The main statistical approach for the MR procedure is the inverse variance weighted (IVW), which is marginally stronger than the other methods [25]. Several additional MR techniques, such as the Weighted median, Weighted mode, MR-egger regression, and MR-PRESSO, were also used to augment the results [26, 27]. Sensitivity analysis was performed using many different methods. First, the heterogeneity of different SNPs was evaluated using the Cochran Q test. If the Cochran Q test was statistically significant, the data showed a substantial degree of heterogeneity. Second, the MR-Egger intercept and MR-PRESSO global tests were employed to determine whether SNPs showed horizontal pleiotropy [27]. No horizontal pleiotropy was found when the p-value of the tests were not significant. Third, to determine whether a specific variant was responsible for the connection between the exposure and outcome variables, the leave-one-out analysis was carried out, in which a specific SNP was eliminated at a time. Funnel plots and forest plots were drawn in our MR analysis to aid in the visual detection of probable horizontal pleiotropy.

We applied the Bonferroni-corrected significance level of *p* < 8.33E − 03 (0.05/6). The 95% confidence interval (CI) for odds ratios (ORs) are used to express estimates of causality. The data were examined using TwoSampleMR (0.5.7) and MRPRESSO (1.0) in R software (Version 4.3.0).

## Results

### Selection of genetic variants

Following the application of stringent criteria to exclude SNPs in the first stage, we screened 57, 51, and 33 significant and independent SNPs for IBD, CD, and UC, respectively (Supplementary Tables 1–3). Seven SNPs were linked to IBD, eight SNPs to CD, and eight SNPs to UC when EN was served as exposure (Supplementary Tables 4–6). No SNPs linked to confounding factors were removed based on the PhenoScanner Database. Moreover, every single SNP that was included had an F-value more than 10, indicating that the likelihood of weak bias was low. The MR-PRESSO analyses revealed no outlier SNPs. Additionally, we found that the use of immunosuppressants did not increase the risk of EN, whereas EN significantly raises the likelihood of immunosuppressants usage (Supplementary Table 7). Notably, the IVs associated with immunosuppressants use did not intersect with those related to EN, as illustrated in Supplementary Table 8. Thus, any potential bias induced by immunosuppressants can be considered insignificant in both the forward and reverse MR analyses.

### Causal effects of IBD and its main subtypes on EN

**Fig. 2:**
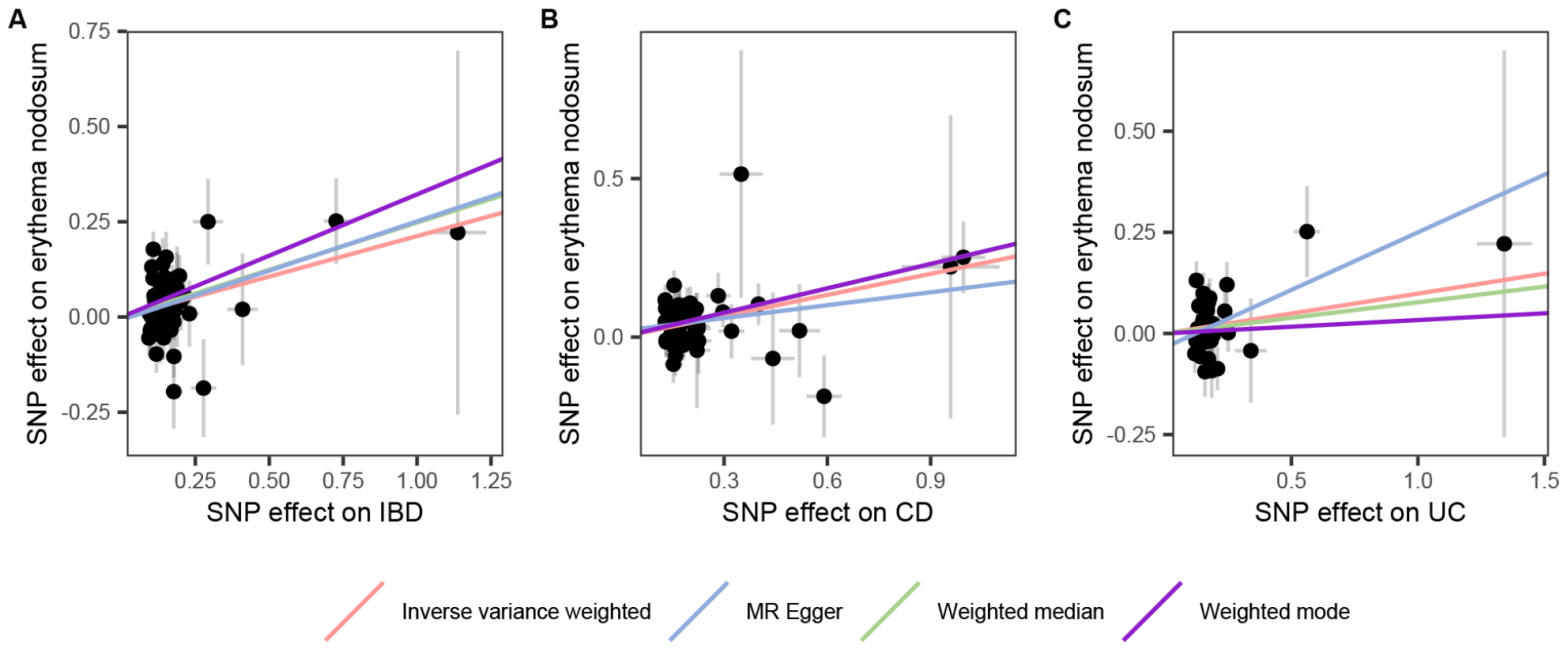
Scatter plots of the forward MR results. The slope of each line corresponds to the MR effect for the respective MR method. (**A**) IBD on EN; (**B**) CD on EN; (**C**) UC on EN. SNP, single-nucleotide polymorphism; IBD, inflammatory bowel disease; EN, erythema nodosum; CD, Crohn’s disease; UC, ulcerative colitis

**Fig. 3:**
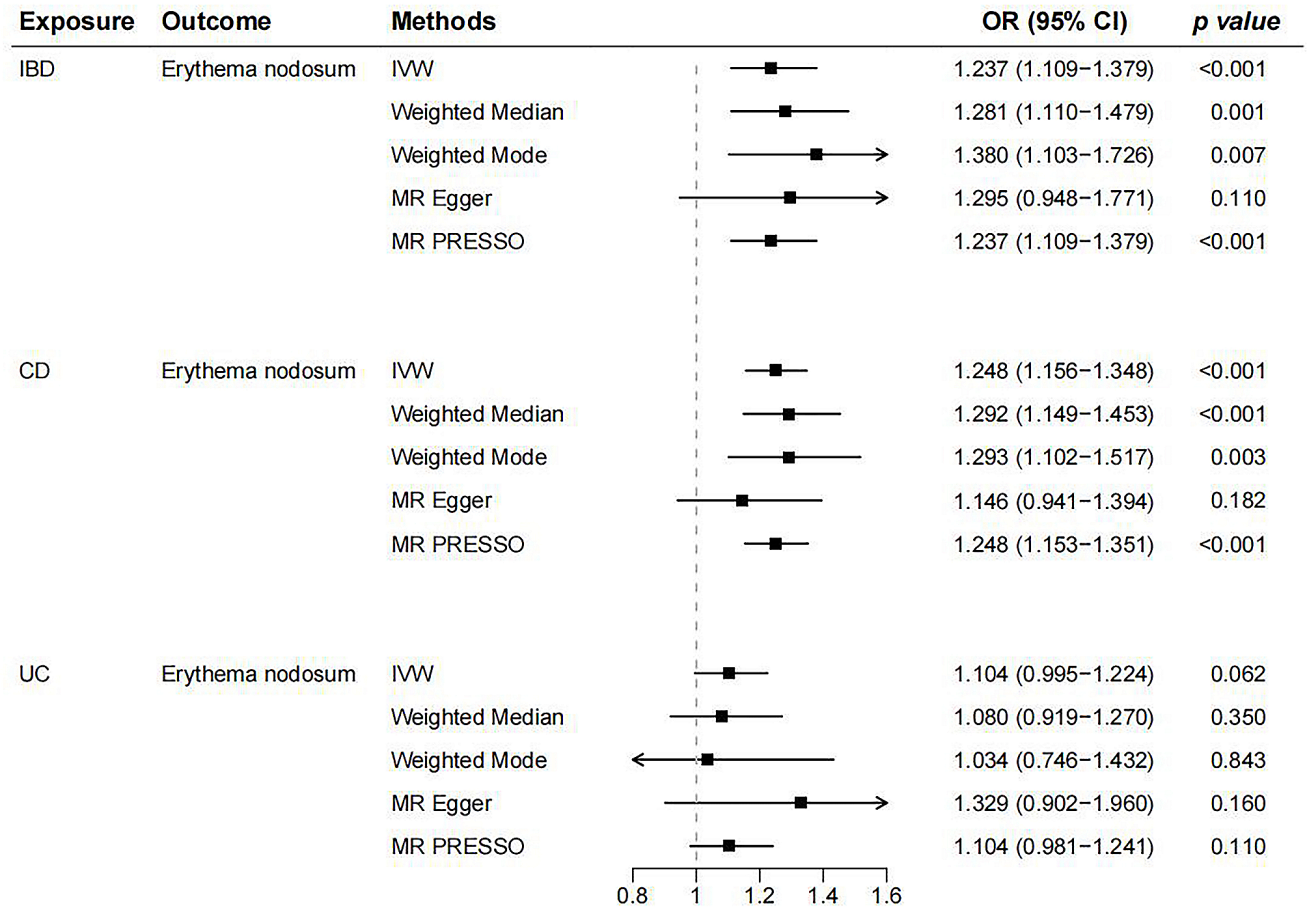
The MR analysis of IBD (CD and UC) on the risk of EN. IBD, inflammatory bowel disease; EN, erythema nodosum; CD, Crohn’s disease; UC, ulcerative colitis; IVW: Inverse variance weighted; OR: Odds Ratio; CI: Confidence Interval; MR-PRESSO, MR‐Pleiotropy Residual Sum and Outlier

In the first stage, the IVW technique demonstrated a strong causality between IBD and EN (OR = 1.237, 95% CI: 1.109–1.37, *p* = 1.43E-08, Fig. 2A). Even after applying the Bonferroni correction, the association remained statistically significant. Additionally, the weighted median (OR = 1.292, 95% CI: 1.149–1.453, *p* = 1.89E-05), weighted mode (OR = 1.380, 95% CI: 1.103–1.726, *p* = 0.007), and MR-PRESSO (OR = 1.237, 95% CI: 1.109 − 1.379, *p* < 0.001) findings matched with the IVW results, although MR Egger did not find a statistically significant difference (OR = 1.295, 95% CI: 0.948–1.771, *p* = 0.110, Fig. 3). In the sensitivity analysis, while MR-Egger regression results (intercept = -0.008, *p* = 0.757) showed that horizontal pleiotropy was unlikely to skew the causality of IBD, the MR-PRESSO global test presented contrasting results with a statistical significance (*p* = 0.044). The Cochran’s Q statistic detected the heterogeneity in the IVs (*p* = 0.038). Furthermore, the leave-one-out analysis showed that this connection was not caused by a single SNP (Supplementary Fig. 1A**)**.

Regarding the two primary subtypes, there was a causal relationship discovered between CD and EN (OR = 0.927, 95% CI: 0.861–0.997, *p* = 0.041, Fig. 2B**)**. The Bonferroni correction test confirmed the causal effect of CD on EN. The results of additional complimentary techniques, in addition to MR-PRESSO, validated the IVW method’s findings (Fig. 3). Sensitivity analyses indicated a lack of significant heterogeneity or horizontal pleiotropy effects in the causal estimates (Supplementary Table 7). The leave-one-out studies confirmed that none of the individual IVs singularly accounted for outcomes (Supplementary Fig. 1B).

However, no causality between UC and EN was identified (OR = 1.104, 95% CI: 0.995 − 1.224, *p* = 0.062, Fig. 2C) and the outcomes of the MR-Egger, MR-PRESSO, Weighted Mode, and Weighted Median analyses agreed with the IVW technique results (Fig. 3). Cochran’s Q statistics, MR-Egger regression, and MR-PRESSO analysis did not show horizontal pleiotropy or heterogeneity in either subgroup of analyses (Supplementary Table **9**). A leave-one-out analysis was performed to further validate the results’ stability and robustness (Supplementary Fig. 1C). Supplementary Figs. 2–3 show the forest plots and funnel plots of SNPs linked with IBD (CD and UC) and EN.

### Causal effects of EN on IBD and its main subtypes

**Fig. 4:**
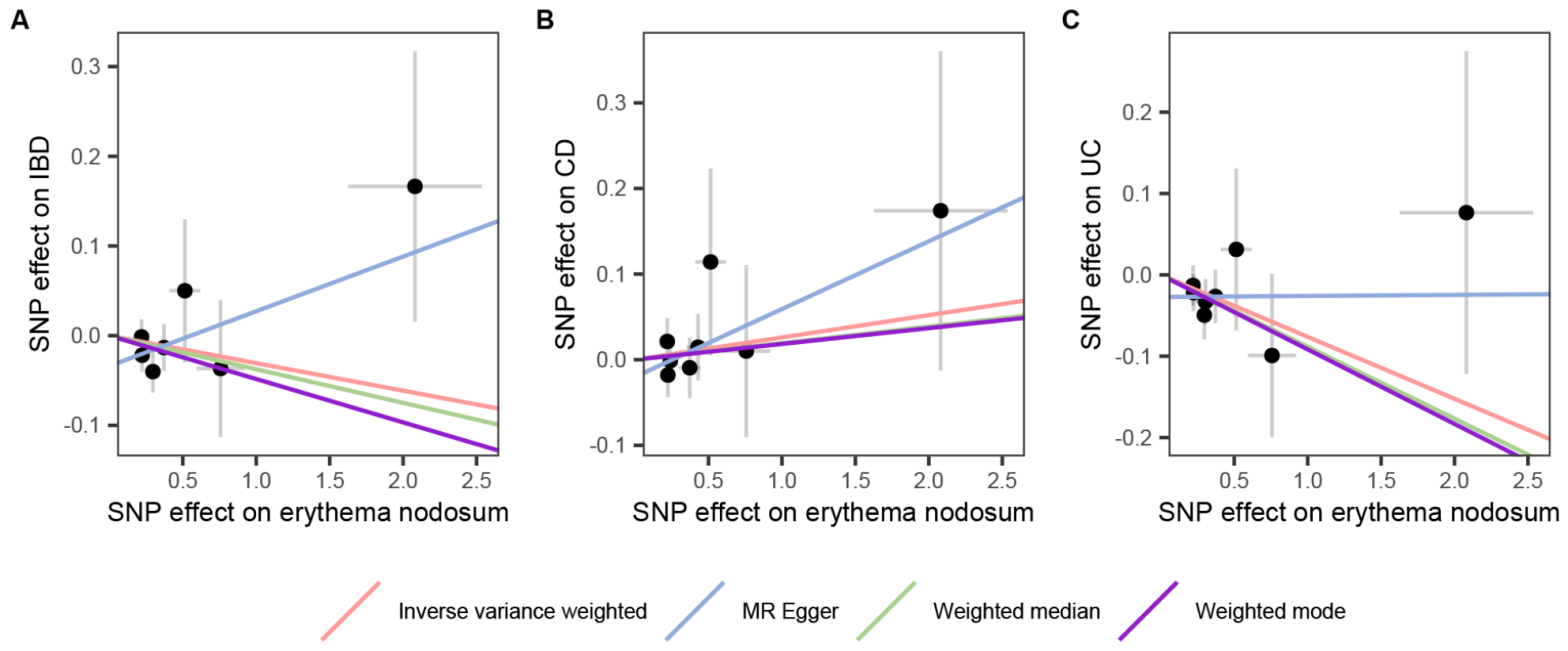
Scatter plots of the reverse MR results. The slope of each line corresponds to the MR effect for the respective MR method. (**A**) EN on IBD; (**B**) EN on CD; (**C**) EN on UC. SNP, single-nucleotide polymorphism; IBD, inflammatory bowel disease; EN, erythema nodosum; CD, Crohn’s disease; UC, ulcerative colitis

**Fig. 5:**
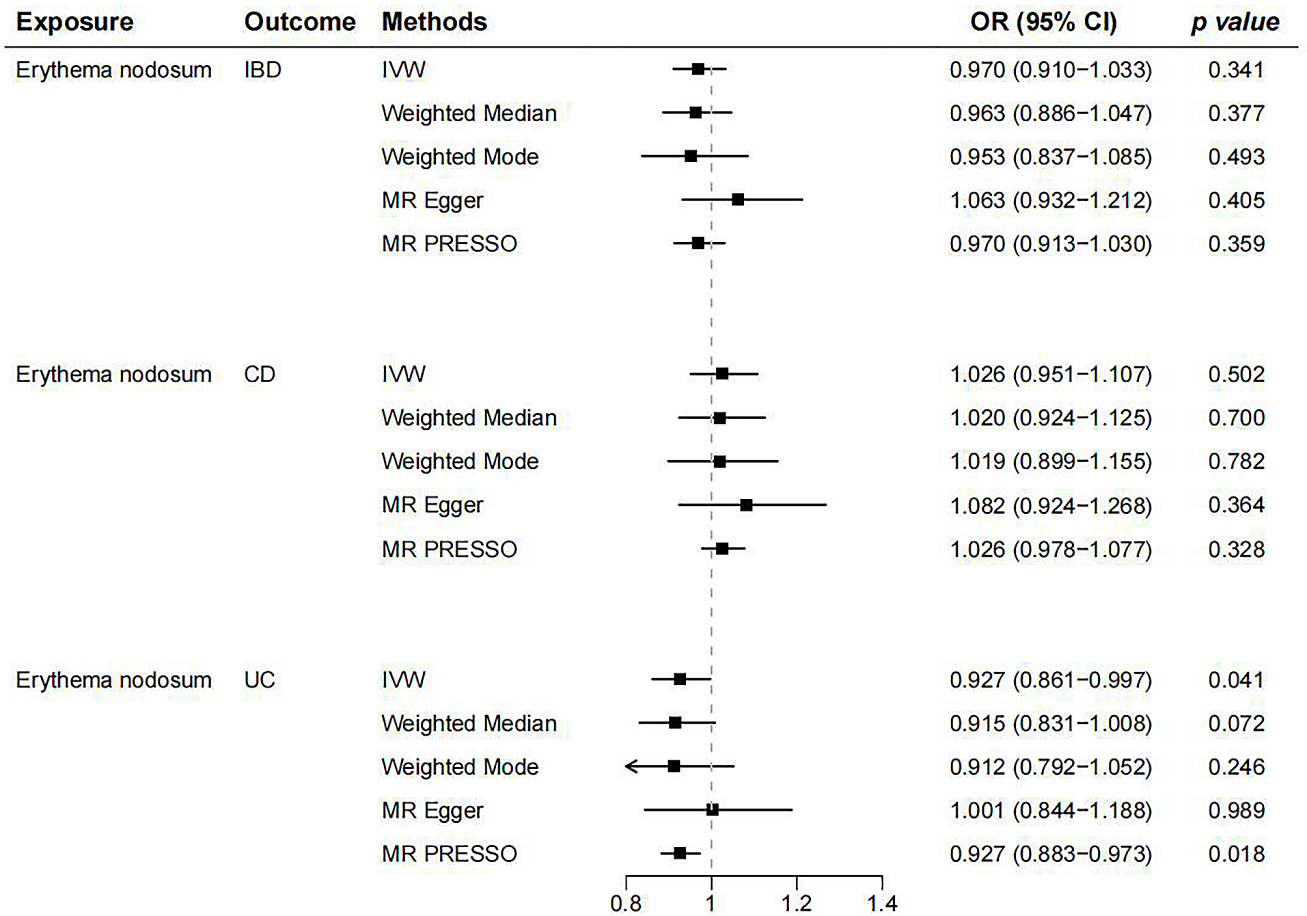
The MR analysis of EN on the risk of IBD (CD and UC). IBD, inflammatory bowel disease; EN, erythema nodosum; CD, Crohn’s disease; UC, ulcerative colitis; IVW: Inverse variance weighted; OR: Odds Ratio; CI: Confidence Interval; MR-PRESSO, MR‐Pleiotropy Residual Sum and Outlier

In the reverse stage, we used EN as exposure data to investigate the causal connection between EN and the danger of IBD and its subtypes. Researchers found no causal association of EN on IBD (OR = 0.970, 95% CI: 0.910 − 1.033, *p* = 0.341, Fig. 4A) and CD (OR = 1.026, 95% CI: 0.951 − 1.107, *p* = 0.502, Fig. 4B) by employing the IVW approach. The results of several additional complementary methods also support this conclusion (Fig. 5). Nonetheless, the IVW technique implied that EN may lower the risk of UC (OR = 0.927, 95% CI: 0.861 − 0.997, *p* = 0.041, Fig. 4C). The MR-PRESSO analyses corroborated the IVW method’s findings, but not MR-Egger regression, weighted median, and weighted mode (Fig. 5). Neither significant heterogeneity nor horizontal pleiotropy were shown by sensitivity analyses (Supplementary Table 9). Furthermore, the causative effect of EN on IBD and its subtypes did not significantly change with any individual SNP during the “leave-one-out” analysis (Supplementary Fig. 4). Scatter plots, forest plots, and funnel plots were shown in Supplementary Figs. 5–6.

## Discussion

In our study, we investigated the causative connection between IBD and EN utilizing the bidirectional MR analysis. The findings demonstrated that total IBD had an increased risk of EN and further demonstrated a causal linkage between EN and CD; however, no such relationship was discovered for UC. In addition, the reverse MR analysis confirmed a negative causal link of EN on UC, but not on CD or IBD.

EN is the most prevalent cutaneous symptom of IBD, and prior epidemiological observations have explored the possibility of a link between the two conditions. However, there is insufficient causal evidence to support this link. The precedence of EN occurrence in CD over UC remains controversial [7, 28–31]. In a prospective cohort research comprising 2,402 individuals with IBD, it was discovered that 5.8% of the patients had at least one cutaneous symptom, with 4% having EN [17]. With a prevalence of 5.6% in CD compared to 1.2% in UC (*p* < 0.001), EN was found to be substantially and independently linked with CD in that study. Another research involving a total of 566 individuals (295 CD and 271 UC) revealed that EN was more common in CD patients (OR = 2.35; 95% CI, 1.13-5.0; *p* = 0.013) [10]. Moreover, an investigation conducted in Turkey also verified that the incidence of EN was higher in CD rather than UC (13.5% vs. 4.2%, *p* = 0.002) [32]. However, a retrospective study utilizing the University of Manitoba IBD Database revealed that EN was similarly present in CD and UC (1.8% vs. 2.2%) [7]. Variations in the rates of EN reporting among patients with IBD between studies reflect the variability of the source populations, recruitment strategies, and data collection methods. Rather, by using EN as an exposure variable, the data show a strong causal relationship between EN and UC. Studies on the order in which EN and IBD occur are limited. 14.3% of patients had an EN diagnosis prior to an IBD diagnosis, according to a large cohort research that assessed the incidence of individuals afflicted by EIM before and after an IBD diagnosis [12]. The capacity of these observational studies to accurately determine causal relationships may be hampered by the unavoidable confounders that influence exposure and outcome. MR analysis may minimize the impact of these confounding factors and generate a relatively precise causal estimates by using genetic instrumental variables.

There is increasing awareness that dermatologic manifestations in IBD patients are related to specific therapies for IBD or consequences resulting from IBD itself. It appears that the occurrence of one EIM raises the risk of acquiring additional ones [8]. However, the proposed mechanism that underlies the correlation between IBD and EN seems yet unclear. Abnormal lymphocyte homing or cross-reactivity, that can lead to the spread of gut disease activity beyond the intestines, may be accountable for the development of EIMs [33]. In addition, it has been proposed that by causing cross-reactions between skin and intestinal antigens, the microbiota-regulated gut-skin axis can affect extraintestinal skin symptoms [34, 35]. Moreover, the role of genetic factors in the link between IBD and EN should not be underestimated. Several potential genetic connections between IBD and EN were linked to HLA region genes, such as HLA-DRB10103, HLA-B27, and HLA-B5 [36]. The genes of SOCS5 (a gene encoding for an inhibitor of cytokines), CD207 (an atypical antigenic pathway gene involved in promoting the uptake of antigens for T cell expression), and ITGB3 (a gene encoding an integrin protein engaged in cell adhesion and surface signal regulation), are involved in the occurrence of EN and the inflammation of IBD [18]. Future research is needed to determine if the various connections between IBD and EN are caused by genetic factors, gut microbiota, or immunological response variations [37–39].

There are various advantages to this study. Firstly, his is the initial study that we are aware of that uses GWAS data to investigate the causal link between IBD and EN. This approach is less prone to reverse causation and confounding factors than observational research. Secondly, to avoid demographic stratification, we limited our study population to people of European heritage. Thirdly, sensitivity analyses were carried out to guarantee dependability of the outcomes and consistency in causal estimates. Simultaneously, our study also has several limitations. To begin with, the MR analysis included only participants of European descent. Ethnicity plays a significant role in the genetic traits associated with IBD and EN, thus caution should be taken when extrapolating the results to a broader population [40, 41]. Additionally, the lack of available GWAS data on the age of onset, disease extent, and various clinical characteristics of IBD prevented us from conducting more detailed stratified analyses using an MR approach. Furthermore, there may be substantial differences in population characteristics between the two samples, such as age, gender, and socioeconomic background, which could affect the validity of causal inference. Lastly, while we explored the potential role of immunosuppressants, we did not individually assess the role of each specific immunosuppressant in the causal relationship between IBD and EN. Specific immunosuppressants could be explored in the future. Our study could provide new insights into genes involved in IBD and EN, but further studies are required to elucidate the precise mechanisms.

## Conclusions

Our MR study results confirmed that IBD and CD have a causal effect on EN, whereas no such effect was shown in UC. EN, in turn, lowers the potential of UC. Healthcare practitioners should maintain heightened vigilance regarding the increased prevalence of EN among IBD patients, especially in those with CD subtype, and implement early diagnostic techniques and specialized treatments accordingly. Further research is needed to dig deeper into the pathophysiological mechanisms driving this association.

### Electronic supplementary material

Below is the link to the electronic supplementary material.

Supplementary Material 1

## Data Availability

The data that support the findings of this study are openly available in IEU GWAS Project at https://gwas.mrcieu.ac.uk/ and FinnGen Consortium at https://r10.finngen.fi/. These data were derived from the following resources available in the public domain: ieu-a-30 (https://gwas.mrcieu.ac.uk/datasets/ieu-a-30/), ieu-a-31 (https://gwas.mrcieu.ac.uk/datasets/ieu-a-31/), ieu-a-32 (https://gwas.mrcieu.ac.uk/datasets/ieu-a-32/), finn-b- L12_ERYTHEMANODOSUM (https://r10.finngen.fi/pheno/L12_ERYTHEMANODOSUM).
