# Supplementary material for: The causal relationship between inflammatory bowel diseases and erythema nodosum: a bidirectional two-sample mendelian randomization study
Source: BMC Gastroenterol. 2024 Jul 23;24:231. doi: 10.1186/s12876-024-03330-8 (PMC11267788; doi:10.1186/s12876-024-03330-8)

| Supplementary Table 1**. Characteristics of genetic variants used to estimate the effect of IBD on EN.** | | | | | | | | | |
| --- | --- | --- | --- | --- | --- | --- | --- | --- | --- |
| **SNP** | **effect allele** | **other allele** | **F** | **Exposure** | | | **Outcome** | | |
|  |  |  |  | **beta** | **se** | **p** | **beta** | **se** | **p** |
| rs1003342 | G | A | 31.977 | -0.095 | 0.017 | 1.67E-08 | -0.009 | 0.047 | 0.851 |
| rs10045431 | C | A | 88.108 | 0.177 | 0.019 | 6.59E-21 | -0.103 | 0.056 | 0.064 |
| rs10408351 | A | G | 38.881 | 0.138 | 0.022 | 4.23E-10 | 0.042 | 0.061 | 0.486 |
| rs10737481 | G | T | 68.893 | 0.141 | 0.017 | 1.19E-16 | 0.002 | 0.047 | 0.970 |
| rs10761659 | G | A | 88.596 | 0.162 | 0.017 | 4.07E-21 | -0.002 | 0.047 | 0.970 |
| rs11209026 | A | G | 296.207 | -0.726 | 0.042 | 1.76E-66 | -0.252 | 0.113 | 0.026 |
| rs11236797 | A | C | 83.888 | 0.156 | 0.017 | 4.75E-20 | -0.025 | 0.048 | 0.601 |
| rs112401990 | A | G | 66.790 | 0.142 | 0.017 | 2.84E-16 | -0.015 | 0.050 | 0.759 |
| rs112694524 | A | G | 38.622 | 0.188 | 0.030 | 5.39E-10 | 0.053 | 0.132 | 0.687 |
| rs11548656 | G | A | 33.351 | -0.293 | 0.051 | 7.72E-09 | -0.250 | 0.112 | 0.026 |
| rs11677953 | A | G | 32.576 | 0.098 | 0.017 | 1.05E-08 | -0.035 | 0.051 | 0.495 |
| rs12446550 | A | G | 39.739 | 0.108 | 0.017 | 2.78E-10 | 0.178 | 0.047 | 0.000 |
| rs1250573 | A | G | 35.752 | -0.114 | 0.019 | 2.21E-09 | -0.009 | 0.049 | 0.852 |
| rs12764283 | A | G | 50.020 | 0.127 | 0.018 | 1.57E-12 | 0.007 | 0.049 | 0.893 |
| rs12936409 | T | C | 75.215 | 0.146 | 0.017 | 3.87E-18 | 0.104 | 0.047 | 0.028 |
| rs13178036 | C | G | 30.073 | 0.101 | 0.018 | 4.13E-08 | 0.001 | 0.051 | 0.979 |
| rs140892874 | C | T | 63.998 | 0.410 | 0.051 | 1.28E-15 | 0.020 | 0.147 | 0.891 |
| rs142770866 | A | G | 46.580 | 0.230 | 0.034 | 8.14E-12 | 0.009 | 0.087 | 0.919 |
| rs148844907 | A | T | 139.524 | 1.138 | 0.096 | 3.63E-32 | 0.222 | 0.478 | 0.643 |
| rs1551399 | C | A | 34.288 | 0.101 | 0.017 | 5.01E-09 | -0.030 | 0.053 | 0.576 |
| rs1736161 | A | G | 50.213 | -0.123 | 0.017 | 1.34E-12 | 0.001 | 0.048 | 0.986 |
| rs17800987 | G | A | 43.733 | 0.202 | 0.031 | 3.71E-11 | 0.042 | 0.078 | 0.587 |
| rs1873625 | A | C | 98.111 | 0.177 | 0.018 | 3.71E-23 | -0.012 | 0.048 | 0.797 |
| rs1887428 | C | G | 92.933 | -0.172 | 0.018 | 6.65E-22 | -0.088 | 0.049 | 0.071 |
| rs2076756 | G | A | 101.722 | 0.188 | 0.019 | 5.59E-24 | 0.104 | 0.066 | 0.113 |
| rs2193041 | G | A | 60.426 | 0.134 | 0.017 | 6.91E-15 | 0.026 | 0.050 | 0.607 |
| rs2241878 | C | T | 76.693 | 0.148 | 0.017 | 1.75E-18 | 0.035 | 0.048 | 0.458 |
| rs2542147 | T | G | 44.425 | -0.151 | 0.023 | 2.78E-11 | -0.157 | 0.067 | 0.019 |
| rs254559 | A | C | 35.721 | 0.103 | 0.017 | 2.08E-09 | 0.131 | 0.047 | 0.005 |
| rs2836882 | A | G | 95.373 | -0.196 | 0.020 | 1.49E-22 | -0.108 | 0.055 | 0.050 |
| rs3024493 | A | C | 92.053 | 0.213 | 0.022 | 8.48E-22 | 0.055 | 0.065 | 0.397 |
| rs3091316 | A | G | 34.691 | -0.112 | 0.019 | 3.59E-09 | -0.043 | 0.050 | 0.384 |
| rs34190331 | A | G | 34.085 | 0.177 | 0.030 | 5.39E-09 | -0.196 | 0.098 | 0.045 |
| rs35260072 | C | A | 69.965 | 0.142 | 0.017 | 7.07E-17 | 0.050 | 0.051 | 0.322 |
| rs35730213 | C | G | 60.908 | -0.151 | 0.019 | 6.91E-15 | -0.058 | 0.058 | 0.318 |
| rs3850378 | C | T | 30.250 | 0.155 | 0.028 | 3.80E-08 | 0.045 | 0.087 | 0.604 |
| rs4077515 | T | C | 108.791 | 0.179 | 0.017 | 1.50E-25 | 0.017 | 0.048 | 0.718 |
| rs4246905 | C | T | 68.462 | 0.163 | 0.020 | 1.42E-16 | 0.061 | 0.059 | 0.304 |
| rs444210 | G | A | 42.484 | 0.110 | 0.017 | 7.39E-11 | -0.013 | 0.047 | 0.789 |
| rs45528737 | T | C | 30.914 | 0.167 | 0.030 | 2.66E-08 | -0.034 | 0.058 | 0.557 |
| rs4676408 | A | G | 42.576 | 0.118 | 0.018 | 6.62E-11 | -0.097 | 0.049 | 0.048 |
| rs4712528 | C | G | 35.077 | 0.123 | 0.021 | 3.07E-09 | 0.044 | 0.053 | 0.410 |
| rs4730272 | G | A | 56.758 | -0.134 | 0.018 | 4.50E-14 | -0.078 | 0.047 | 0.101 |
| rs56062135 | T | C | 58.083 | 0.151 | 0.020 | 2.64E-14 | 0.057 | 0.054 | 0.288 |
| rs6062496 | A | G | 84.025 | 0.165 | 0.018 | 5.48E-20 | 0.024 | 0.049 | 0.628 |
| rs6584283 | C | T | 113.824 | -0.180 | 0.017 | 1.70E-26 | -0.053 | 0.047 | 0.262 |
| rs6826501 | T | C | 30.149 | -0.093 | 0.017 | 4.12E-08 | 0.055 | 0.049 | 0.258 |
| rs6873866 | C | T | 36.958 | -0.107 | 0.018 | 1.09E-09 | -0.101 | 0.049 | 0.038 |
| rs6880778 | G | A | 117.843 | 0.188 | 0.017 | 2.14E-27 | 0.075 | 0.048 | 0.115 |
| rs6911490 | C | T | 47.134 | -0.143 | 0.021 | 6.82E-12 | 0.055 | 0.059 | 0.352 |
| rs6927172 | G | C | 29.813 | 0.110 | 0.020 | 4.65E-08 | 0.056 | 0.060 | 0.355 |
| rs72798422 | C | T | 41.484 | 0.278 | 0.043 | 1.19E-10 | -0.187 | 0.129 | 0.147 |
| rs7285952 | G | T | 56.092 | -0.176 | 0.024 | 7.60E-14 | -0.035 | 0.074 | 0.634 |
| rs744166 | G | A | 49.247 | -0.121 | 0.017 | 2.16E-12 | -0.049 | 0.048 | 0.306 |
| rs7523335 | A | G | 38.995 | -0.141 | 0.023 | 4.16E-10 | 0.021 | 0.053 | 0.695 |
| rs9370774 | C | T | 35.620 | -0.131 | 0.022 | 2.54E-09 | 0.046 | 0.075 | 0.538 |
| rs9934775 | T | C | 36.205 | -0.140 | 0.023 | 1.71E-09 | -0.139 | 0.068 | 0.040 |

IBD, inflammatory bowel disease; EN, erythema nodosum; SNP: single-nucleotide polymorphisms.

**Supplementary Table 2**. Characteristics of genetic variants used to estimate the effect of CD on EN.

| **SNP** | **effect allele** | **other allele** | **F** | **Exposure** | | | **Outcome** | | |
| --- | --- | --- | --- | --- | --- | --- | --- | --- | --- |
|  |  |  |  | **beta** | **se** | **p** | **beta** | **se** | **p** |
| rs1056441 | C | T | 42.890 | 0.167 | 0.026 | 5.44E-11 | 0.014 | 0.054 | 0.798 |
| rs10748781 | A | C | 84.750 | -0.219 | 0.024 | 3.72E-20 | -0.089 | 0.050 | 0.074 |
| rs10761659 | G | A | 80.020 | 0.212 | 0.024 | 3.42E-19 | -0.002 | 0.047 | 0.970 |
| rs11209026 | A | G | 242.559 | -0.995 | 0.064 | 1.05E-54 | -0.252 | 0.113 | 0.026 |
| rs11236797 | A | C | 61.466 | 0.181 | 0.023 | 4.85E-15 | -0.025 | 0.048 | 0.601 |
| rs112401990 | A | G | 31.116 | 0.132 | 0.024 | 2.35E-08 | -0.015 | 0.050 | 0.759 |
| rs114607072 | T | G | 49.334 | 0.442 | 0.063 | 2.20E-12 | -0.067 | 0.208 | 0.746 |
| rs11564236 | T | A | 76.113 | 0.519 | 0.060 | 2.85E-18 | 0.020 | 0.147 | 0.890 |
| rs12194825 | A | T | 33.277 | -0.172 | 0.030 | 8.00E-09 | -0.049 | 0.056 | 0.385 |
| rs1250573 | A | G | 41.904 | -0.171 | 0.026 | 9.01E-11 | -0.009 | 0.049 | 0.852 |
| rs12717899 | T | G | 30.344 | 0.159 | 0.029 | 3.59E-08 | -0.058 | 0.064 | 0.361 |
| rs1297271 | T | C | 42.718 | -0.155 | 0.024 | 6.28E-11 | 0.001 | 0.048 | 0.984 |
| rs13135092 | G | A | 32.421 | 0.221 | 0.039 | 1.21E-08 | -0.041 | 0.182 | 0.820 |
| rs140054334 | T | C | 31.025 | 0.350 | 0.063 | 2.57E-08 | 0.514 | 0.390 | 0.187 |
| rs1456896 | T | C | 30.801 | 0.139 | 0.025 | 2.90E-08 | 0.091 | 0.052 | 0.082 |
| rs147018773 | T | C | 73.593 | 0.322 | 0.038 | 8.89E-18 | 0.019 | 0.086 | 0.825 |
| rs147684209 | C | T | 40.302 | 0.155 | 0.024 | 2.34E-10 | 0.163 | 0.047 | 0.001 |
| rs148844907 | A | T | 45.579 | 0.958 | 0.142 | 1.47E-11 | 0.222 | 0.478 | 0.643 |
| rs151314883 | A | G | 46.922 | -0.224 | 0.033 | 7.12E-12 | -0.036 | 0.074 | 0.625 |
| rs1873625 | A | C | 55.300 | 0.181 | 0.024 | 1.09E-13 | -0.012 | 0.048 | 0.797 |
| rs1887428 | C | G | 47.854 | -0.168 | 0.024 | 4.22E-12 | -0.088 | 0.049 | 0.071 |
| rs1932990 | T | C | 33.799 | 0.153 | 0.026 | 6.02E-09 | -0.086 | 0.059 | 0.146 |
| rs2076756 | G | A | 272.940 | 0.400 | 0.024 | 3.24E-61 | 0.104 | 0.066 | 0.113 |
| rs2129944 | G | T | 33.222 | -0.156 | 0.027 | 7.81E-09 | -0.050 | 0.052 | 0.332 |
| rs2188962 | T | C | 86.782 | 0.212 | 0.023 | 1.36E-20 | 0.077 | 0.051 | 0.128 |
| rs2505640 | G | A | 37.794 | -0.146 | 0.024 | 7.61E-10 | -0.006 | 0.049 | 0.897 |
| rs281379 | A | G | 34.502 | 0.140 | 0.024 | 4.26E-09 | 0.072 | 0.048 | 0.133 |
| rs28701841 | A | G | 36.162 | 0.224 | 0.037 | 1.85E-09 | 0.028 | 0.083 | 0.742 |
| rs3024505 | A | G | 34.702 | 0.178 | 0.030 | 3.90E-09 | 0.054 | 0.065 | 0.406 |
| rs3091315 | G | A | 46.582 | -0.180 | 0.026 | 9.52E-12 | -0.046 | 0.050 | 0.353 |
| rs3810936 | C | T | 62.427 | 0.208 | 0.026 | 2.46E-15 | 0.066 | 0.056 | 0.238 |
| rs4077515 | T | C | 84.406 | 0.216 | 0.024 | 4.37E-20 | 0.017 | 0.048 | 0.718 |
| rs444210 | G | A | 50.915 | 0.163 | 0.023 | 1.02E-12 | -0.013 | 0.047 | 0.789 |
| rs4820091 | G | T | 37.073 | 0.172 | 0.028 | 1.22E-09 | 0.099 | 0.051 | 0.053 |
| rs4851586 | C | T | 41.877 | -0.169 | 0.026 | 9.94E-11 | -0.066 | 0.060 | 0.269 |
| rs4902642 | A | G | 29.970 | -0.129 | 0.024 | 4.34E-08 | -0.117 | 0.047 | 0.014 |
| rs56062135 | T | C | 51.528 | 0.193 | 0.027 | 7.45E-13 | 0.057 | 0.054 | 0.288 |
| rs6588243 | C | A | 31.679 | 0.132 | 0.023 | 1.78E-08 | -0.010 | 0.047 | 0.833 |
| rs6704109 | T | C | 62.263 | 0.202 | 0.026 | 2.77E-15 | 0.107 | 0.052 | 0.041 |
| rs6873866 | C | T | 49.467 | -0.168 | 0.024 | 2.06E-12 | -0.101 | 0.049 | 0.038 |
| rs697693 | A | G | 37.596 | 0.172 | 0.028 | 8.36E-10 | -0.014 | 0.079 | 0.860 |
| rs72798422 | C | T | 135.068 | 0.590 | 0.051 | 3.19E-31 | -0.187 | 0.129 | 0.147 |
| rs744166 | G | A | 30.795 | -0.129 | 0.023 | 2.92E-08 | -0.049 | 0.048 | 0.306 |
| rs7543234 | T | C | 33.918 | 0.155 | 0.027 | 6.10E-09 | -0.031 | 0.052 | 0.555 |
| rs7713270 | T | C | 151.466 | 0.297 | 0.024 | 6.97E-35 | 0.079 | 0.048 | 0.098 |
| rs7714401 | A | T | 42.679 | 0.159 | 0.024 | 6.20E-11 | 0.090 | 0.051 | 0.075 |
| rs78487399 | C | G | 37.276 | 0.226 | 0.037 | 1.03E-09 | -0.012 | 0.103 | 0.907 |
| rs80262450 | A | G | 64.319 | 0.283 | 0.035 | 1.08E-15 | 0.131 | 0.073 | 0.072 |
| rs8178977 | C | G | 49.512 | 0.193 | 0.027 | 2.06E-12 | 0.102 | 0.054 | 0.059 |
| rs907092 | A | G | 32.708 | 0.130 | 0.023 | 1.01E-08 | 0.091 | 0.047 | 0.055 |
| rs921720 | G | A | 47.241 | 0.163 | 0.024 | 6.40E-12 | -0.027 | 0.053 | 0.615 |

Beta: SNP effect of effect allele; SE, standard error of SNP effect; SNP: single-nucleotide polymorphisms.

**Supplementary Table 3.** Characteristics of genetic variants used to estimate the effect of UC on EN.

| **SNP** | **effect allele** | **other allele** | **F** | **Exposure** | | | **Outcome** | | |
| --- | --- | --- | --- | --- | --- | --- | --- | --- | --- |
|  |  |  |  | **beta** | **se** | **p** | **beta** | **se** | **p** |
| rs10182512 | A | G | 51.995 | 0.161 | 0.022 | 5.19E-13 | -0.021 | 0.050 | 0.679 |
| rs10272963 | T | C | 63.338 | -0.172 | 0.022 | 1.69E-15 | 0.063 | 0.047 | 0.186 |
| rs10737481 | G | T | 134.071 | 0.250 | 0.022 | 4.37E-31 | 0.002 | 0.047 | 0.970 |
| rs10917545 | A | G | 30.530 | -0.185 | 0.034 | 3.29E-08 | 0.092 | 0.067 | 0.172 |
| rs11209026 | A | G | 118.039 | -0.562 | 0.052 | 1.58E-27 | -0.252 | 0.113 | 0.026 |
| rs114152040 | A | G | 29.714 | 0.340 | 0.062 | 4.95E-08 | -0.043 | 0.129 | 0.741 |
| rs12612675 | G | A | 31.494 | 0.123 | 0.022 | 1.98E-08 | -0.018 | 0.051 | 0.721 |
| rs12817473 | G | A | 77.230 | 0.191 | 0.022 | 1.71E-18 | 0.024 | 0.050 | 0.635 |
| rs1359946 | A | G | 34.631 | 0.158 | 0.027 | 3.84E-09 | -0.094 | 0.062 | 0.128 |
| rs137845 | G | A | 31.085 | 0.118 | 0.021 | 2.38E-08 | -0.050 | 0.048 | 0.296 |
| rs148844907 | A | T | 151.704 | 1.341 | 0.109 | 7.17E-35 | 0.222 | 0.478 | 0.643 |
| rs1801274 | G | A | 71.038 | -0.183 | 0.022 | 3.78E-17 | -0.017 | 0.047 | 0.719 |
| rs1886731 | C | T | 40.418 | -0.141 | 0.022 | 2.25E-10 | 0.057 | 0.047 | 0.229 |
| rs1887428 | C | G | 62.227 | -0.177 | 0.022 | 3.36E-15 | -0.088 | 0.049 | 0.071 |
| rs2212434 | T | C | 44.381 | 0.142 | 0.021 | 2.46E-11 | -0.022 | 0.048 | 0.648 |
| rs254559 | A | C | 33.427 | 0.124 | 0.022 | 7.63E-09 | 0.131 | 0.047 | 0.005 |
| rs3024493 | A | C | 73.299 | 0.236 | 0.028 | 1.09E-17 | 0.055 | 0.065 | 0.397 |
| rs35730213 | C | G | 46.462 | -0.167 | 0.025 | 8.81E-12 | -0.058 | 0.058 | 0.318 |
| rs3829111 | A | G | 53.347 | 0.156 | 0.021 | 2.89E-13 | 0.008 | 0.048 | 0.861 |
| rs4574921 | T | C | 34.607 | 0.151 | 0.026 | 4.24E-09 | 0.033 | 0.060 | 0.583 |
| rs4676410 | A | G | 53.538 | 0.208 | 0.028 | 2.46E-13 | -0.087 | 0.053 | 0.101 |
| rs483905 | A | G | 31.964 | 0.129 | 0.023 | 1.57E-08 | 0.003 | 0.058 | 0.954 |
| rs484356 | G | C | 34.644 | -0.134 | 0.023 | 3.95E-09 | -0.068 | 0.048 | 0.159 |
| rs56167332 | A | C | 43.068 | 0.152 | 0.023 | 5.30E-11 | 0.099 | 0.051 | 0.054 |
| rs6017342 | C | A | 63.538 | 0.191 | 0.024 | 1.38E-15 | -0.004 | 0.048 | 0.941 |
| rs6062496 | A | G | 50.067 | 0.158 | 0.022 | 1.47E-12 | 0.024 | 0.049 | 0.628 |
| rs6933404 | C | T | 43.811 | 0.167 | 0.025 | 3.68E-11 | 0.057 | 0.060 | 0.346 |
| rs7752873 | T | C | 36.197 | 0.182 | 0.030 | 1.83E-09 | -0.018 | 0.077 | 0.816 |
| rs7911680 | C | A | 65.056 | -0.172 | 0.021 | 8.27E-16 | -0.076 | 0.047 | 0.106 |
| rs798502 | C | A | 32.617 | -0.136 | 0.024 | 1.21E-08 | 0.024 | 0.050 | 0.626 |
| rs9823546 | A | T | 62.928 | 0.177 | 0.022 | 2.29E-15 | -0.006 | 0.048 | 0.906 |
| rs989960 | T | C | 36.054 | -0.129 | 0.022 | 1.77E-09 | -0.012 | 0.047 | 0.795 |
| rs9977672 | A | G | 88.120 | -0.245 | 0.026 | 6.21E-21 | -0.121 | 0.056 | 0.031 |

Beta: SNP effect of effect allele; SE, standard error of SNP effect; SNP: single-nucleotide polymorphisms.

**Supplementary Table 4.** Characteristics of genetic variants used to estimate the effect of EN on IBD.

| **SNP** | | **effect allele** | | **other allele** | | **F** | **Exposure** | | | | | **Outcome** | | | | | |  |  |
| --- | --- | --- | --- | --- | --- | --- | --- | --- | --- | --- | --- | --- | --- | --- | --- | --- | --- | --- | --- |
|  | |  | |  | |  | **beta** | **se** | | **p** | | **beta** | | **se** | | **p** | |  |  |
| rs1218838 | | A | | G | | 21.039 | -0.219 | 0.048 | | 4.50E-06 | | 0.001 | | 0.020 | | 0.942 | |  |  |
| rs1340821 | | C | | T | | 24.595 | -0.372 | 0.075 | | 7.07E-07 | | 0.013 | | 0.026 | | 0.609 | |  |  |
| rs144719519 | | A | | G | | 20.947 | 2.081 | 0.455 | | 4.72E-06 | | 0.166 | | 0.151 | | 0.270 | |  |  |
| rs186880954 | | C | | G | | 21.605 | 0.757 | 0.163 | | 3.35E-06 | | -0.037 | | 0.077 | | 0.632 | |  |  |
| rs6074423 | | C | | T | | 21.315 | -0.223 | 0.048 | | 3.90E-06 | | 0.022 | | 0.018 | | 0.236 | |  |  |
| rs74362947 | | T | | A | | 24.050 | 0.514 | 0.105 | | 9.39E-07 | | 0.050 | | 0.080 | | 0.530 | |  |  |
| rs798895 | | T | | C | | 40.630 | -0.296 | 0.047 | | 1.84E-10 | | 0.040 | | 0.023 | | 0.086 | |  |  |
| **Table S5.** Characteristics of genetic variants used to estimate the effect of EN onCD. | | | | | | | | | | | | | | | | | | | |
| **SNP** | | **effect allele** | | **other allele** | | **F** | | **Exposure** | | | | | | **Outcome** | | | | | |
|  | |  | |  | |  | | **beta** | | **se** | | **p** | | **beta** | | **se** | | **p** | |
| rs113010081 | | C | | T | | 31.160 | | -0.429 | | 0.077 | | 2.38E-08 | | -0.015 | | 0.039 | | 0.709 | |
| rs1218838 | | A | | G | | 21.039 | | -0.219 | | 0.048 | | 4.50E-06 | | -0.021 | | 0.027 | | 0.431 | |
| rs1340821 | | C | | T | | 24.595 | | -0.372 | | 0.075 | | 7.07E-07 | | 0.009 | | 0.035 | | 0.791 | |
| rs144719519 | | A | | G | | 20.947 | | 2.081 | | 0.455 | | 4.72E-06 | | 0.174 | | 0.186 | | 0.351 | |
| rs186880954 | | C | | G | | 21.605 | | 0.757 | | 0.163 | | 3.35E-06 | | 0.010 | | 0.101 | | 0.920 | |
| rs2099684 | | G | | A | | 22.807 | | 0.240 | | 0.050 | | 1.79E-06 | | -0.001 | | 0.025 | | 0.962 | |
| rs6074423 | | C | | T | | 21.315 | | -0.223 | | 0.048 | | 3.90E-06 | | 0.018 | | 0.026 | | 0.488 | |
| rs74362947 | | T | | A | | 24.050 | | 0.514 | | 0.105 | | 9.39E-07 | | 0.114 | | 0.109 | | 0.295 | |

**Supplementaey Table 6**. Characteristics of genetic variants used to estimate the effect of EN on UC.

| **SNP** | **effect allele** | **other allele** | **F** | **Exposure** | | | **Outcome** | | |
| --- | --- | --- | --- | --- | --- | --- | --- | --- | --- |
|  |  |  |  | **beta** | **se** | **p** | **beta** | **se** | **p** |
| rs1218838 | A | G | 21.039 | -0.219 | 0.048 | 4.50E-06 | 0.013 | 0.025 | 0.598 |
| rs1340821 | C | T | 24.595 | -0.372 | 0.075 | 7.07E-07 | 0.026 | 0.033 | 0.418 |
| rs144719519 | A | G | 20.947 | 2.081 | 0.455 | 4.72E-06 | 0.076 | 0.199 | 0.700 |
| rs186880954 | C | G | 21.605 | 0.757 | 0.163 | 3.35E-06 | -0.099 | 0.100 | 0.323 |
| rs6074423 | C | T | 21.315 | -0.223 | 0.048 | 3.90E-06 | 0.022 | 0.023 | 0.338 |
| rs74362947 | T | A | 24.050 | 0.514 | 0.105 | 9.39E-07 | 0.031 | 0.099 | 0.753 |
| rs75506426 | C | T | 21.274 | -0.305 | 0.066 | 3.98E-06 | 0.033 | 0.028 | 0.237 |
| rs798895 | T | C | 40.630 | -0.296 | 0.047 | 1.84E-10 | 0.049 | 0.030 | 0.096 |

**Supplementary Table 7. MR results of immunosuppressants and EN.**

| **Exposure** | **Outcome** | **Method** | **No. SNP** | **OR** | **95% CI** | **p value** |
| --- | --- | --- | --- | --- | --- | --- |
| Immunosuppressants | EN | IVW | 5 | 1.004 | 0.771-1.309 | 0.974 |
|  |  | Weighted Median | 5 | 0.972 | 0.792-1.193 | 0.784 |
|  |  | MR Egger | 5 | 0.867 | 0.457-1.645 | 0.692 |
|  |  | Weighted mode | 5 | 0.931 | 0.732-1.183 | 0.588 |
|  |  | MR_PRESSO | 5 | 1.004 | 0.771-1.309 | 0.975 |
| EN | Immunosuppressants | IVW | 9 | 1.086 | 1.013-1.165 | 0.020 |
|  |  | Weighted Median | 9 | 1.074 | 0.974-1.185 | 0.152 |
|  |  | MR Egger | 9 | 1.041 | 0.762-1.422 | 0.810 |
|  |  | Weighted mode | 9 | 1.089 | 0.916-1.295 | 0.363 |
|  |  | MR_PRESSO | 9 | 1.086 | 0.996-1.185 | 0.099 |

EN, erythema nodosum; SNP, single-nucleotide polymorphism; OR, odds ratio; CI, confidence interval; IVW, inverse variance weighted; MR‐PRESSO, MR‐Pleiotropy Residual Sum and Outlier.

**Supplementary Table 8.** Characteristics of genetic variants used to estimate the effect of EN on immunosuppressants.

| **SNP** | **effect allele** | **other allele** | **F** | **Exposure** | | | **Outcome** | | |
| --- | --- | --- | --- | --- | --- | --- | --- | --- | --- |
|  |  |  |  | **beta** | **se** | **p** | **beta** | **se** | **p** |
| rs143236495 | A | G | 30.308 | 0.381 | 0.069 | 3.70E-08 | -0.105 | 0.097 | 0.277 |
| rs2507996 | A | G | 41.882 | -0.154 | 0.024 | 9.70E-11 | 0.073 | 0.054 | 0.177 |
| rs6679677 | A | C | 80.513 | 0.334 | 0.037 | 2.90E-19 | 0.088 | 0.067 | 0.187 |
| rs687308 | T | C | 378.921 | 0.561 | 0.029 | 2.10E-84 | -0.048 | 0.067 | 0.467 |
| rs9276609 | G | C | 65.605 | 0.189 | 0.023 | 5.50E-16 | 0.105 | 0.049 | 0.032 |

EN, erythema nodosum; SNP, single-nucleotide polymorphism.

**Supplementary Table 9. Sensitivity analysis of the present study.**

|  |  |  | **Cochran Q test** | | **MR-Egger Pleiotropy test** | | **MR PRESSO (outlier-corrected)** | | |
| --- | --- | --- | --- | --- | --- | --- | --- | --- | --- |
| **Exposure** | **Outcome** | **SNPs** | **Q** | **P value** | **Intercept** | **P value** | **outlier** | **OR (95% CI)** | **p for global test** |
| IBD | EN | 57 | 76.181 | 0.038 | -0.008 | 0.757 | None | 1.237 (1.109-1.379) | 0.044 |
| CD | EN | 51 | 53.267 | 0.350 | 0.019 | 0.353 | None | 1.248 (1.153-1.351) | 0.377 |
| UC | EN | 33 | 41.359 | 0.124 | -0.035 | 0.331 | None | 1.104 (0.981-1.241) | 0.126 |
| EN | IBD | 7 | 5.536 | 0.477 | -0.034 | 0.180 | None | 0.970 (0.913-1.030) | 0.473 |
| EN | CD | 8 | 2.844 | 0.899 | -0.020 | 0.481 | None | 1.026 (0.978-1.077) | 0.903 |
| EN | UC | 7 | 3.083 | 0.877 | -0.027 | 0.364 | None | 0.927 (0.883-0.973) | 0.860 |

MR, Mendelian randomization analysis; SNPs, Number of single nucleotide polymorphism; IBD, inflammatory bowel disease; CD, Crohn's disease; UC, ulcerative colitis; EN, erythema nodosum; OR: Odds Ratio; CI: Confidence Interval; MR-PRESSO, MR-Pleiotropy Residual Sum and Outlier.

**Supplementary Figure 1**. Leave-one-out analysis of IBD (A), CD (B), and UC (C) on EN. IBD, inflammatory bowel disease; EN, erythema nodosum; CD, Crohn's disease; UC, ulcerative colitis.


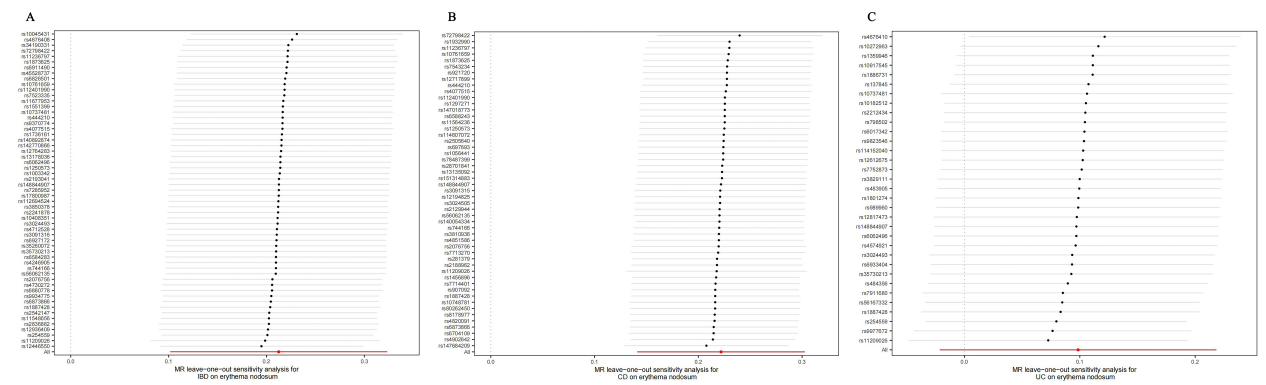


**Supplementary Figure 2**. Forest plot of SNPs associated with IBD (A), CD (B), and UC (C) on the risk of EN. IBD, inflammatory bowel disease; EN, erythema nodosum; CD, Crohn's disease; UC, ulcerative colitis.


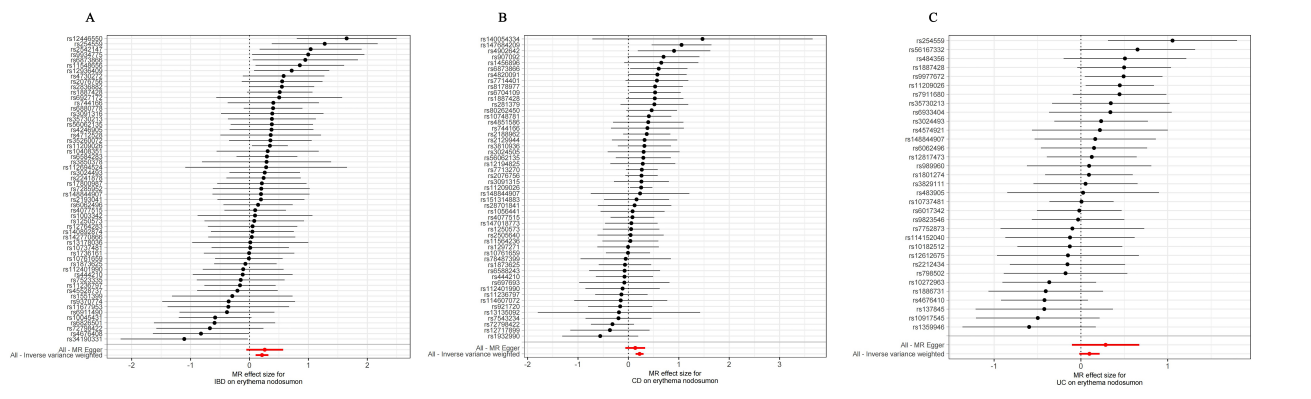


**Supplementary Figure 3**. Funnel plot of SNPs associated with IBD (A), CD (B), and UC (C) on the risk of EN.IBD, inflammatory bowel disease; EN, erythema nodosum; CD, Crohn's disease; UC, ulcerative colitis.


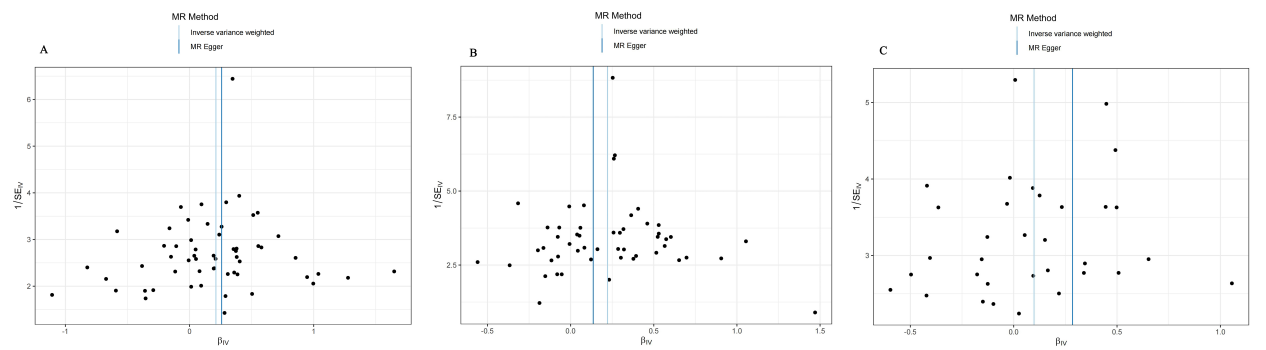


**Supplementary Figure 4**. Leave-one-out analysis of EN on IBD (A), CD (B), and UC (C). IBD, inflammatory bowel disease; EN, erythema nodosum; CD, Crohn's disease; UC, ulcerative colitis.


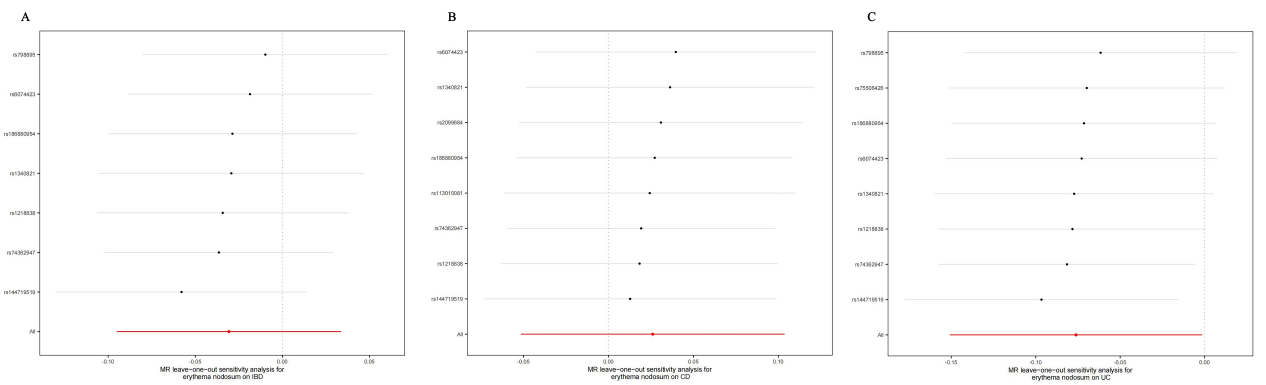


**Supplementary Figure 5**. Forest plot of SNPs associated with EN on the risk of IBD (A), CD (B), and UC (C). IBD, inflammatory bowel disease; EN, erythema nodosum; CD, Crohn's disease; UC, ulcerative colitis.


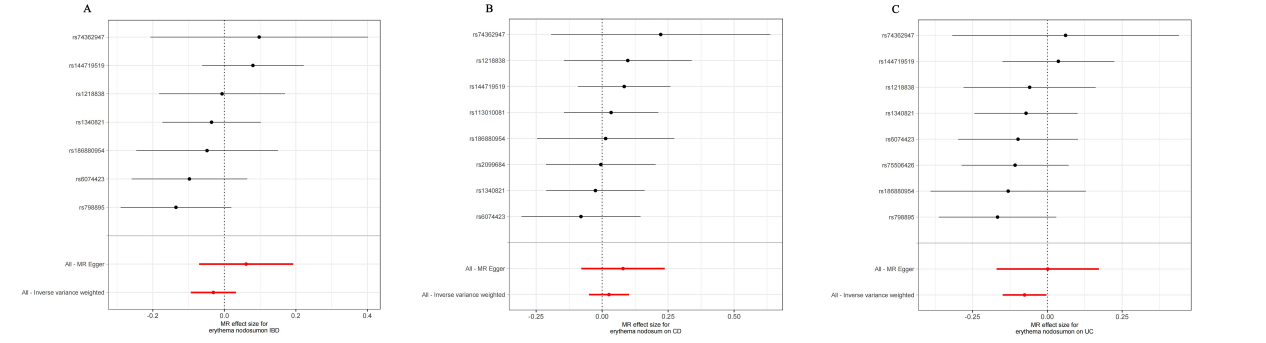


**Supplementary Figure 6**. Funnel plot of SNPs associated with EN on the risk of IBD (A), CD (B), and UC (C). IBD, inflammatory bowel disease; EN, erythema nodosum; CD, Crohn's disease; UC, ulcerative colitis.


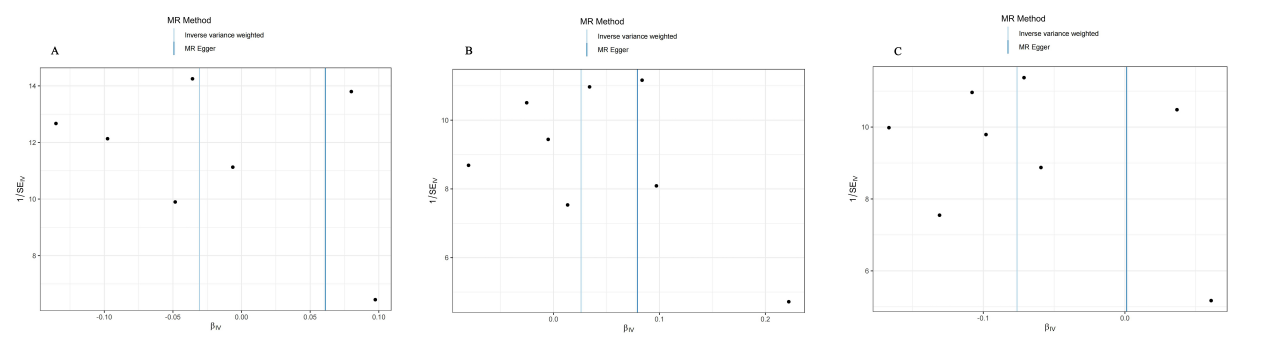

Supplement: Supplementary file 1 — Supplementary Material 1 [file 12876_2024_3330_MOESM1_ESM.docx]
